# Retroperitoneal Bronchogenic Cyst: A Rare Case Study

**DOI:** 10.7759/cureus.10421

**Published:** 2020-09-13

**Authors:** Vikash Sinha, Partha Nandi, Manu Shankar, Nitin Sardana

**Affiliations:** 1 General Surgery, Fortis Escorts Hospital, Faridabad, IND

**Keywords:** bronchogenic cyst

## Abstract

Bronchogenic cyst usually presents along the tracheobronchial tree. Rarely, it is found inside peritoneal cavity. Here is a case of 30-year-old man who presented with concerns of abdominal pain. On evaluation, contrast-enhanced CT scan showed hypodense cystic lesion in epigastric region related to right crus of diaphragm. The patient underwent laparoscopic marsupialization/deroofing of cyst. Histopathological examination of resected specimen showed respiratory epithelium. Postoperative period was uneventful.

## Introduction

Bronchogenic cyst is an abnormal developmental anomaly of foregut which is usually present in mediastinum. Occasionally, it is found at unusual locations, such as retroperitoneum. Microscopically, it is lined with ciliated pseudostratified columnar epithelium, and its wall contains fibrous elastic tissue with normal bronchial elements. Mostly it is asymptomatic, but sometimes it may get infected or produces compressive symptoms [1]. Here is a case of subdiaphragmatic retroperitoneal bronchogenic cyst diagnosed after histopathological analysis of a resected specimen.

## Case presentation

A 30-year-old gentleman from Faridabad, India, presented with concerns of upper abdominal pain along with a sense of discomfort for last one day, which was sudden in onset, progressive, and radiating towards back. There was no prior history of similar event. No other specific complaints were noted.

**Figure 1:**
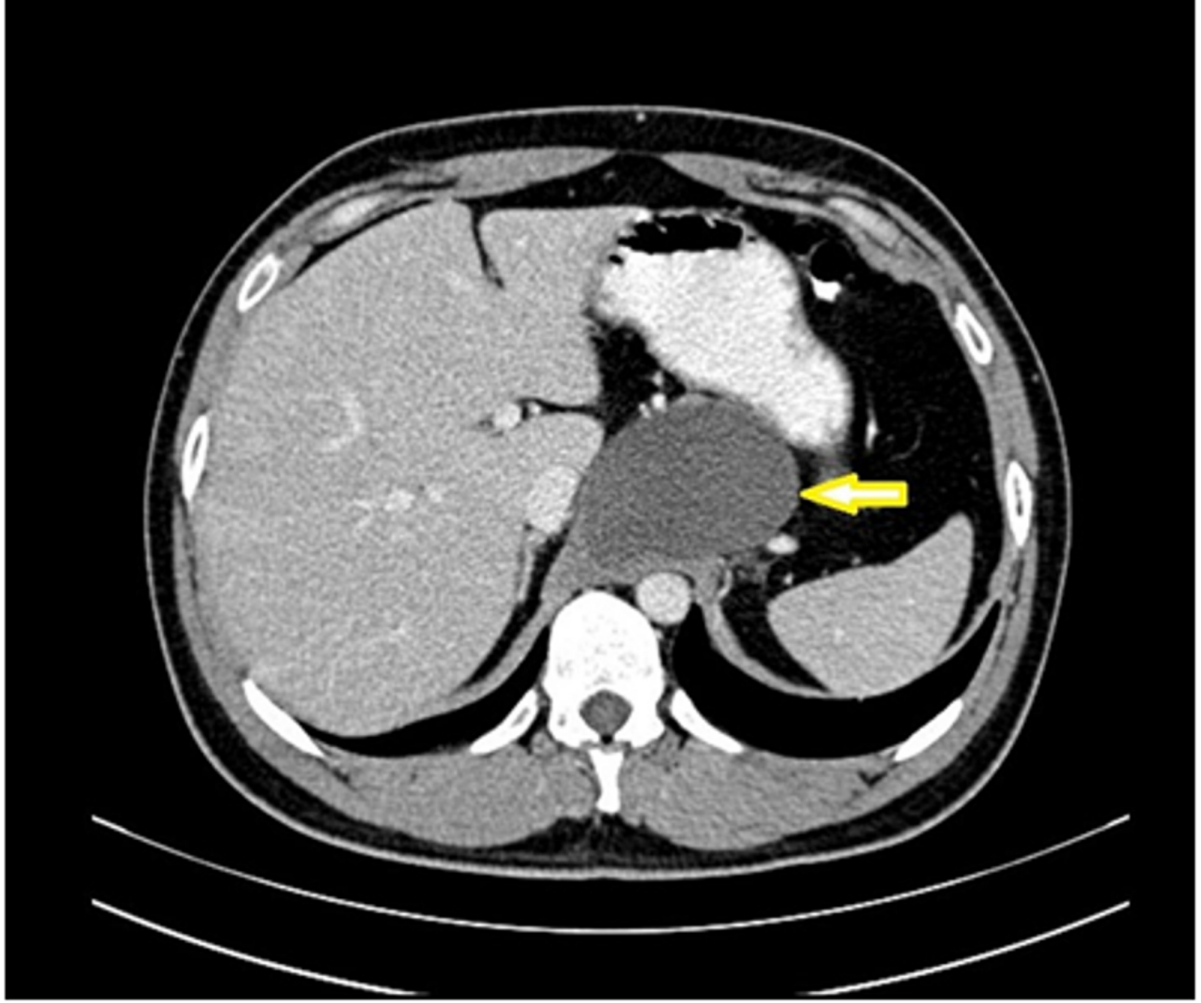
A cut section from contrast-enhanced CT whole abdomen showing the location of the cyst (arrowhead)

On examination, his vitals were normal. Abdomen was soft, non-distended and tenderness was present over epigastric and supra umbilical area. There was no palpable lump or organomegaly nor any thrill could be appreciated. Ultrasonography of whole abdomen showed a well-defined hypoechoic lesion with internal echoes (5.9 × 4.2 × 4.2 cm) in pre-aortic region in the epigastrium with no definite intralesional color flow on Doppler. Contrast-enhanced CT (CECT) scan of whole abdomen revealed a well-defined thin-walled homogeneous hypodense cystic lesion (70 × 45 mm) in epigastric region related to right crus of diaphragm near the midline. The lesion was indenting the stomach and abutting the adjacent liver, pancreas, left adrenal, inferior vena cava, aorta, and celiac axis (Figure 1). Based on radiological findings, a diagnosis of bronchogenic cyst with differential diagnosis of diaphragmatic and gastric duplication cyst was made.

**Figure 2:**
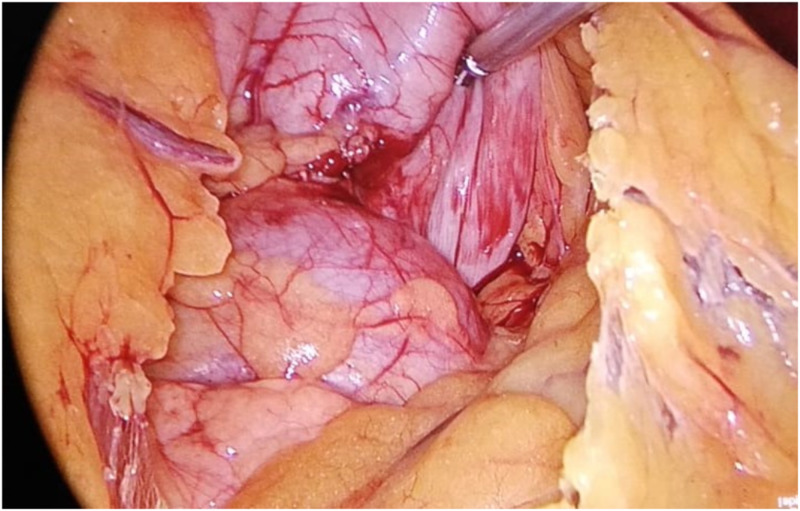
Laparoscopic view of bronchogenic cyst

After obtaining an informed consent, laparoscopic marsupialization/deroofing was done. Complete excision could not be achieved due to dense posterior adhesion to abdominal aorta and inferior vena cava. Intraoperatively, a 7 × 5 cm large well-defined cystic lesion was present in the midline in the retroperitoneum having both true and false capsules and containing approximately 100 ml of white colored fluid. Relations of the cyst are as follows: anterior relation - posterior surface of stomach and body of pancreas, posterior relation - abdominal aorta and inferior vena cava, on the right side - lesser omentum with right gastric vessels. on the left side - posterior surface of stomach, superiorly related to inferior surface of diaphragm and inferiorly to the body of pancreas (Figure 2).

**Figure 3:**
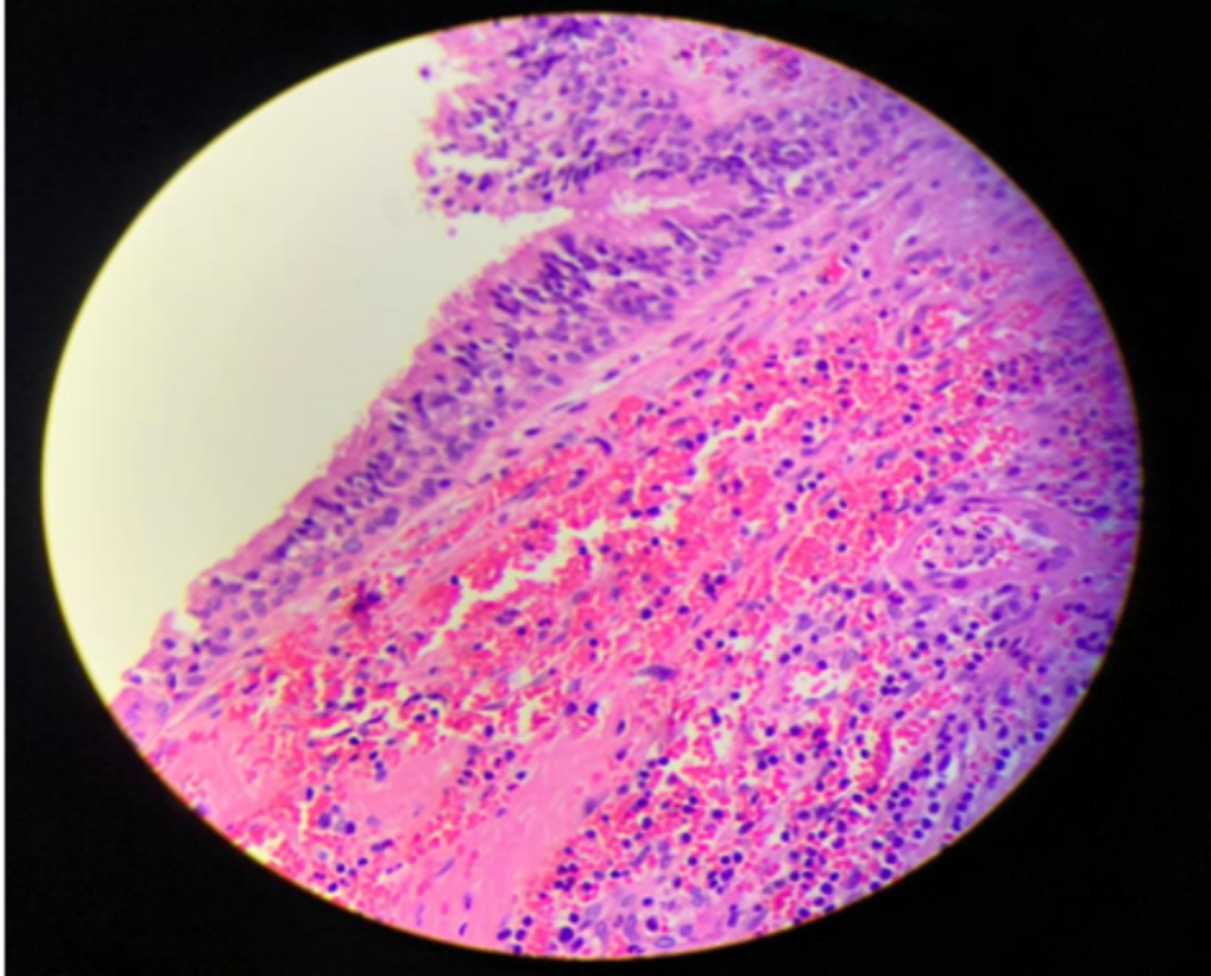
Microscopic view of wall of retroperitoneal bronchogenic cyst showing characteristic respiratory epithelium (hematoxylin and eosin stain, ×400)

Histopathological examination revealed specimen lined with respiratory epithelium (ciliated columnar epithelium) (Figure 3).

Postoperative period was uneventful, and he was managed conservatively and recovered well.

## Discussion

During early embryonic life, tracheobronchial analge of primitive foregut sometimes gives an abnormal budding which becomes bronchogenic cyst later in life. When its attachment to primitive foregut persists, it is associated with tracheobronchial tree or esophagus [1].

Retroperitoneal bronchogenic cyst is rare. During early embryonic life, pericardioperitoneal canal links thoracic and abdominal cavity. When the canal is obliterated by the pleuroperitoneal membrane, a part of tracheobronchial tree pinched-off and migrate, resulting in the development of bronchogenic cyst [2].

Most of the retroperitoneal bronchogenic cysts found in the superior border of pancreas or near the left adrenal gland. Most of the patients are asymptomatic, and the cyst is found incidentally on radiological investigations. Symptomatic patients present with epigastric or back pain. Most of reported cases are less than 5 cm in size, and the largest reported case is 10 cm in size in which patient presented with pheochromocytoma like symptoms due to external compression of cyst and subsequent release of catecholamines [3].

In 2004, Kim et al. reported a very unusual presentation of bronchogenic cyst which was located at inferior surface of liver and clinically mimicked as gallbladder tumor [4].

In 2015, Trehan et al. reported a case of brochogenic cyst in the right side of retropeitoneum in Ludhiana, India. The patient presented with right flank pain. CECT revealed a large cyst in the right hypochondrium. Excision of the cyst was done, and histopathological examination showed bronchogenic cyst [5].

In 2020, Bakshi et al. reported a case of bronchogenic cyst in lesser sac with intermediate grade neuroendocrine tumor arising from its wall. Hydatid cyst of liver was also concomitantly present in this patient [6].

In 2002, Sullivan et al. reported a case of adenocarcinoma arising in a retroperitoneal bronchogenic cyst in a 55-year-old female [7].

CT and MRI are the mainstay of diagnostic modality. CT scan of bronchogenic cyst typically shows sharply marginated masses of soft tissue or water attenuation. Most of them appears cystic. Few lesions appear solid and can be confused with other lesions. MRI can also be used to elucidate the cystic nature of disease [8].

Treatment of bronchogenic cyst is surgical excision. Majority of cysts are asymptomatic and benign in nature. Surgical excision is done to confirm the diagnosis, alleviate the symptoms, and prevent the future risk of infection and malignant transformation [7].

## Conclusions

Retroperitoneal bronchogenic cyst is a rare entity. Mostly, it is asymptomatic and found incidentally on CT or MRI scan. It should always be kept in mind as differential diagnosis of retroperitoneal cyst, Treatment is laparoscopic excision of cyst. 
